# Alfalfa *MsbHLH115* confers tolerance to cadmium stress through activating the iron deficiency response in *Arabidopsis thaliana*

**DOI:** 10.3389/fpls.2024.1358673

**Published:** 2024-02-12

**Authors:** Miao Zhang, Jing-Yun Gao, Shi-Chen Dong, Meng-Han Chang, Jing-Xuan Zhu, Dong-Lin Guo, Chang-Hong Guo, Ying-Dong Bi

**Affiliations:** ^1^ Heilongjiang Provincial Key Laboratory of Molecular Cell Genetics and Genetic Breeding, College of Life Science and Technology, Harbin Normal University, Harbin, China; ^2^ Institute of Crops Tillage and Cultivation, Heilongjiang Academy of Agricultural Sciences, Harbin, China

**Keywords:** cadmium, iron nutrient, MsbHLH115, transcription factors, alfalfa

## Abstract

Cadmium (Cd) pollution severely affects plant growth and development, posing risks to human health throughout the food chain. Improved iron (Fe) nutrients could mitigate Cd toxicity in plants, but the regulatory network involving Cd and Fe interplay remains unresolved. Here, a transcription factor gene of alfalfa, *MsbHLH115* was verified to respond to iron deficiency and Cd stress. Overexpression of *MsbHLH115* enhanced tolerance to Cd stress, showing better growth and less ROS accumulation in *Arabidopsis thaliana*. Overexpression of *MsbHLH115* significantly enhanced Fe and Zn accumulation and did not affect Cd, Mn, and Cu concentration in Arabidopsis. Further investigations revealed that *MsbHLH115* up-regulated iron homeostasis regulation genes, ROS-related genes, and metal chelation and detoxification genes, contributing to attenuating Cd toxicity. Y1H, EMSA, and LUC assays confirmed the physical interaction between MsbHLH115 and E-box, which is present in the promoter regions of most of the above-mentioned iron homeostasis regulatory genes. The transient expression experiment showed that MsbHLH115 interacted with *MsbHLH121pro*. The results suggest that *MsbHLH115* may directly regulate the iron-deficiency response system and indirectly regulate the metal detoxification response mechanism, thereby enhancing plant Cd tolerance. In summary, enhancing iron accumulation through transcription factor regulation holds promise for improving plant tolerance to Cd toxicity, and *MsbHLH115* is a potential candidate for addressing Cd toxicity issues.

## Graphical Abstract

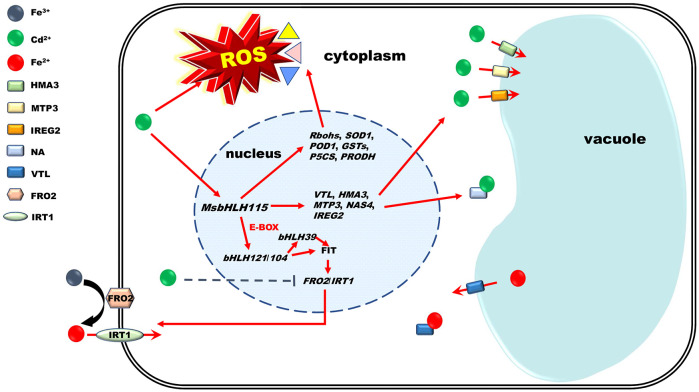

## 1 Introduction

Cadmium (Cd) is a biologically non-essential metal and one of the most hazardous environmental pollutants. Cd contamination in crops has become a severe problem due to rapid industrialization and excessive use of pesticides and fertilizers (Palansooriya et al., 2020). Cd injures plant growth and reproduction, disrupts photosynthesis and transpiration, impairs root growth, and reduces biomass production by catalyzing the accumulation of harmful substances (Sandalio et al., 2001; Dias et al., 2012; El Rasafi et al., 2020). Most economic crops are sensitive to Cd toxicity, and high Cd accumulation poses a potential risk to food safety. Therefore, enhancing Cd tolerance and reducing Cd accumulation in edible parts of crops is of great significance.

Iron (Fe) is an essential metal element for plants that plays a role in plant growth and development. Because Fe deficiency hurts plant growth and excess Fe is toxic to plants, a set of transcript factors and transporters tightly regulates Fe homeostasis. Due to the high similarity in ionic hydrated radius of Cd^2+^ (4.26 am) and Fe^2+^ (4.28 am) (Nightingale, 1959), Cd can be easily absorbed and transported by competing with Fe (Eide et al., 1996). The crosstalk between Cd and Fe has been found in plants. Cd stress changed the cell wall components and enhanced the binding capacity for Fe, resulting in Fe retention in the apoplast of roots and suppressing Fe translocation from roots to shoots (Xu et al., 2015). The antagonistic interaction between Cd and Fe may contribute to the Cd toxicity symptoms observed in plants (Jian et al., 2019), and Fe deficiency can exacerbate Cd toxicity in plants (Bao et al., 2009; Muneer et al., 2014; Su et al., 2014), while elevating the Fe status reduces the Cd toxicity to plants and alleviate Cd toxicity symptoms, contributing to enhancing the tolerance to Cd stress (Meda et al., 2007; Liu et al., 2020).

Cd and be uptake by identified Fe transporters. The intracellular vesicle membrane protein iron-regulated transporter1 (IRT1) was found to be involved in Cd uptake (Korshunova et al., 1999; Zhang et al., 2020). It has been reported that limiting Fe uptake through the downregulation of Fe acquisition mechanisms confers Si-mediated alleviation of Cd toxicity in Alfalfa (Kabir et al., 2016). Results indicated that argon-stimulated NO production contributes to Cd tolerance by transcriptional reduction in representative target genes involved in heavy metal detoxification, antioxidant defiance, and iron homeostasis (Wang J. et al., 2023). To avoid heavy metal poisoning, plants often up-regulate the expression of genes related to metal chelation, sequestration, and redeployment, including *heavy metal-associated domain* (*HMA*), *Metal tolerance protein* (*MTP*), *Iron regulatory gene* (*IREG*), and other metal transporters (Yao et al., 2018; Sheng et al., 2019; Dang et al., 2022). The conjugation and sequestration of Cd into vacuoles by glutathione and proline are also essential mechanisms promoting Cd tolerance in plants (Xiang et al., 2001; Cai et al., 2011; Koen et al., 2013; Han et al., 2020). Exogenous CH4 mitigates Cd toxicity in alfalfa by inducing miR159 and miR167 to regulate heavy metal transporters, reduce Cd accumulation, and reconstruct glutathione homeostasis (Gu et al., 2018). Although several genes in the synthesis and decomposition of glutathione and proline have been identified, the regulating mechanism of glutathione and proline under Cd stress remains unclear.

Members of TF including *WRKY*, *MYB*, *NAC*, *bHLH*, and *MYC* are recognized to enhance Cd tolerance by regulating genes expression in a variety of plants (Sheng et al., 2019; Zhang et al., 2019; Du et al., 2023; Han et al., 2023; Wang B. et al., 2023). Several studies have shown that TFs can regulate genes to improve Fe accumulation and distribution, significantly reducing the toxicity of Cd to plants (Zhang et al., 2019; Shi et al., 2023). bHLH TFs have received increasing attention for their roles in plant development, stress response, and organ communication (Aparicio and Pallás, 2016). Their parts in response networks against heavy metal stress have also been highlighted (Yadav and Mani, 2019). *IRT1* is directly controlled by *FIT* and its interacting partners *bHLH38*/*39* in Fe uptaking (Colangelo and Guerinot, 2004; Schwarz and Bauer, 2020; Riaz and Guerinot, 2021; Liang, 2022). It has been reported that overexpression of *FIT* with *AtbHLH38* or *AtbHLH39* enhances Cd tolerance in Arabidopsis by increasing root Cd uptake and improving shoot Fe homeostasis (Wu et al., 2012).

The bHLH IVc genes are widely expressed in plants and have been shown to function as long-distance organ-to-organ signals from shoots to roots, participating in Fe uptake, translocation, and intracellular homeostasis (Grillet et al., 2018). *bHLH104* is also responsive to Cd toxicity, and mutant plants lacking *bHLH104* are sensitive to Cd stress, while overexpression of *bHLH104* increases Cd tolerance by altering Fe translocation (Zhang et al., 2015; Li et al., 2016; Yao et al., 2018; Tissot et al., 2019; Zhou et al., 2019). BTS interacts with bHLH104 and negatively regulates Fe homeostasis at the post-translational level, significantly increasing tolerance to Cd stress and rising Fe and Cd accumulation (Zhu et al., 2020). The ectopic expression of *IMA* peptides confers Cd tolerance to Arabidopsis by activating Fe-deficiency responses (Meng X. et al., 2022). These studies suggest that bHLH IVc genes are involved in the crosstalk of Fe and Cd. Previously, it was found that the expression of *IMAs* is regulated by *bHLH121*, and their expression is highly induced in *bhlh121* mutants (Li et al., 2022). Studies suggest that the expression of bHLH IVc may be regulated by bHLH121. Chip assays have demonstrated that *bHLH115* regulates downstream *bHLH121*, *FIT*, *bHLH38*/*39*/*100*/*101*, and *POPEYE* (Li et al., 2016; Liang et al., 2017; Gao et al., 2020). The mechanism by which *bHLH115* mediates Cd tolerance in plants remains elusive (Kurt et al., 2019; Lei et al., 2020; Lockhart, 2020). It is unclear whether *bHLH115*-mediated Fe accumulation can confer Cd tolerance in these overexpressing lines, which could provide new solutions for improving tolerance to Cd toxicity. Activating the Fe-deficiency regulatory system will effectively enhance plant tolerance to Cd (He et al., 2017; Ai et al., 2022). However, the underlying mechanism remains unclear due to a lack of general systemic signaling across species for Fe deficiency regulation and an insufficient understanding of the antagonistic mechanism between Fe and Cd.

*Medicago sativa* (alfalfa) is an important forage grass (Ndayambaza et al., 2020). Cd adversely affects alfalfa growth(Kabir et al., 2016). In this study, we explored the function of *MsbHLH115* in Arabidopsis and determined that *MsbHLH115* regulates plant Cd tolerance. Our results suggest that *MsbHLH115* may regulate plant Cd tolerance by controlling the expression of iron-regulated transcription factors bHLHs, which increases iron transport. We identified *MsbHLH115* as a regulator of Cd tolerance, indicating that *MsbHLH115* could be used to design new strategies for crop adaptation to Cd stress.

## 2 Materials and methods

### 2.1 Plant materials and stress treatments

Alfalfa (*Medicago sativa* cv. Zhao dong) seeds, provided by the Livestock and Veterinary Research Institute of Heilongjiang Academy of Agricultural Sciences, were germinated in distilled water for 3 days. Seedlings were cultivated in Hoagland’s solution (10 μM MnSO_4_, 100 μM H_3_BO_3_, 0.1 μM CuSO_4_, 0.1 μM Na_2_MoO_4_, 30 μM ZnSO_4_, 5 μM KI, 0.1 μM CoCl_2_, 4 mM CaCl_2_, 1 mM MgSO_4_, 1 mM KH_2_PO_4_, 100 μM Fe(III)-EDTA and 5 mM KNO_3_, pH 5.8) at 25°C, 16 h light/8 h darkness (Cui et al., 2013). The culture solution was changed every 3 days. Four-week-old alfalfa seedlings were selected for treatment. Alfalfa seedlings were treated with +Cd stress for 3 days to analyze the expression of iron-deficiency response genes. The stress concentration of hydroponics in Hoagland’s solution was −Fe (0 µM Fe-EDTA+0 μM CdCl_2_), +Cd (100 µM Fe-EDTA+90 μM CdCl_2_), −Fe+Cd (0 µM Fe-EDTA+90 μM CdCl_2_), and the control (CK, 100 µM Fe-EDTA+0 μM CdCl_2_) (Dai et al., 2017; Yang et al., 2021). Alfalfa seedlings were treated with −Fe, +Cd, −Fe+Cd, and CK for 24 h, and the roots, stems, and leaves were collected, respectively, to analyze the tissue-specific expression of *MsbHLH115*. Alfalfa seedlings were treated with 50 μM, 70 μM, 90 μM, 100 μM, and 200 μM Cd for 24 h to detect the response of *MsbHLH115*. Alfalfa seedlings were treated with −Fe, +Cd, −Fe+Cd, and CK for 6, 12, 24, 48, and 72 h to detect the temporal properties of *MsbHLH115* expression. The above samples were collected, frozen in liquid nitrogen, and stored at −80°C to extract total RNA for Quantitative Real-Time Polymerase Chain Reaction (qRT-PCR).

The cultivation method of Arabidopsis seeds (ecotype Columbia, Col-0) is as follows: surface-sterilized with 10% NaClO for 15 min and then washed six times with distilled water, seeds were plated on 1/2 MS medium (Murashige and Skoog, 1962) with 1% sucrose, 0.8% agar, and 100 µM Fe-EDTA at pH 5.8, cultivated at 22°C, 16 h light/8 h darkness. The Arabidopsis seedlings were treated with −Fe, +Cd, −Fe+Cd, and CK for phenotype observation and physiological detection.

### 2.2 qRT-PCR

qRT-PCR was performed by the Bio-Rad CFX96 detection system (Bio-Rad, Hercules, CA, USA) using the Top Green qPCR Super Mix (Transgenic Biotechnology Company, Beijing, China). The relative expression levels of genes were calculated using the 2^-ΔΔCT^ comparative method as described by (Livak and Schmittgen, 2001). *MsACTIN2* (Wang et al., 2015) and *AtACTIN2* (AT3G18780) (Czechowski et al., 2005) were used as internal reference genes. The Arabidopsis genes *bHLH115*, *bHLH104*, *bHLH121*, *FIT*, *bHLH39*, *FRO2*, *IRT1*, *VTL*, *HMA3*, *MTP3*, *NAS4*, *IREG2*, *SOD1*, *POD1*, *RbohB, RbohD, RbohF, BGLU14*, *GSTL1*/*2*/*4*/*6*, *AtP5CS*, and *AtPRODH* were detected in the transgenic Arabidopsis and WT under 90 μM CdCl_2_ for 24 h. Primers used in this study were designed using Primer 5.0 software (http://www.premierbiosoft.com/primerdesign/) and online NCBI Primer-BLAST (http://www.ncbi.nlm.nih.gov/tools/primer-blast/), and listed in **Supplementary Table S1**.

### 2.3 Gene isolation and bioinformatics analysis

Total RNA was extracted using the Total RNA Extraction Kit (Omega BioTek, Norcross, GA, USA). Then, RNA was reverse transcribed to cDNA using ReverTra Ace^®^qPCR RT Master Mix with gDNA Remover (TOYOBO, Japan). Total DNA was extracted using the Total DNA Extraction Kit (OMGAE, USA). Primers are shown in **Supplementary Table S1**. The PCR products were cloned into the pMD18-T vector (Takara) and sequenced (Sangon, Shanghai, China). Sequence alignment was performed using DANMAN software. The structured domain was obtained through the SMART (HTTP://smart.embl-heidelberg.de/ accessed on 9 June 2022). Phylogenetic analysis was performed using the MEGA 7.0 with neighbor-joining and a bootstrap repeat value of 1000 times. The cis-elements were analyzed using PlantCare (http://bioinformatics.psb.ugent.be/webtools/plantcare/html/ accessed on 9 June 2022). Finally, TBtools was used to splice.

### 2.4 Subcellular localization of MsbHLH115

*MsbHLH115* without the stop codon was inserted into the pBWA(V)HS vector (provided by BioRun Biotechnology Co., Ltd, Wuhan, China), fused with the green fluorescent protein (GFP) driven by the 35S promoter. The recombinational pBWA(V)HS-35S::*MsbHLH115*-Glosgfp plasmid was transformed into Arabidopsis protoplasts through polyethylene glycol treatment (Yoo et al., 2007). nuclear localization of the MsbHLH115-GFP fusion protein. Red fluorescent protein (RFP) is used as a nuclear localization marker. Fluorescence signals were visualized after 12-16 h using a confocal laser-scanning microscope (Olympus FluoView FV1000, Olympus, Tokyo, Japan).

### 2.5 GUS assay

The *MsbHLH115* promoter fragment was digested with *Bgl* II and *BamH* I to construct a pBI121-*MsbHLH115pro*::GUS and transformed into *Agrobacterium rhizogenes* K599 by the freeze-thaw method. Soybean (*Glycine max* cv. Williams 82) hairy roots were induced by needle insertion of K599 (Kereszt et al., 2007). The soybean complex was cultivated in 1/2 Hoagland’s solution for 7 days. The hairy roots were divided into two parts and treated with −Fe, +Cd, −Fe+Cd, and CK for 24 h, respectively (Li et al., 2023). Histochemical staining for GUS was conducted following the method described previously (Li et al., 2023). The photograph was taken using a stereomicroscope (EZ4-HD LEICA, Germany) coupled with a color charge-coupled device (CCD) camera (Zeiss, Germany). The GUS enzyme activity was detected using a plant β-glucuronidase GUS ELISA kit (Coolaber, SL7160) and enzyme-labeler machine (Ferdi Bio, FlexA-200). The absorbance (OD value) was measured at 450 nm wavelength. The GUS enzyme activity was calculated according to the standard curve previously drawn.

### 2.6 Generation of transgenic plants and phenotypic observation

The *MsbHLH115* gene was digested with *Xba* I and *BamH* I and inserted into pBI121. The recombinant vector pBI121-35S:: *MsbHLH115* was introduced into *Agrobacterium tumefacient* GV3101 by freeze-thaw method and then transformed into Arabidopsis by the filter-dipping process (Clough and Bent, 1998). PCR and qRT-PCR confirmed the *MsbHLH115* transgenic Arabidopsis. The primers used are listed in **Supplementary Table S1**. The *MsbHLH115* transgenic Arabidopsis produced offspring by self-breeding. T_3_ seedlings of *MsbHLH115* transgenic Arabidopsis and WT were cultivated in 1/2 MS medium for 5 days. The growth of *MsbHLH115*ox lines and WT plants were treated with different combinations of Fe and Cd treatments for 7 days. The growth of *#12-2* plants was compared when different root parts were in the split medium with combinations of Fe and Cd treatments for 7 days. A root-tip elongation experiment was applied on *MsbHLH115*ox lines and WT. The plants were planted on a split medium and were treated with different combinations of Fe and Cd treatments. The part near the root tip (RT) and the part distal the root tip (RS) were exposed to different treatments for 7 days. Phenotypic photographs were taken, and root length and fresh weight were recorded.

### 2.7 Physiological index determination

Several previous studies have reported that *bHLH115* plays a role in iron homeostasis. In this study, we performed experiments focused on Cd treatment rather than iron deficiency treatment to better explain its role in Cd tolerance, The Arabidopsis leaves were collected and frozen at −80°C for physiological index determination. The total chlorophyll content was measured according to the method described by (Yavari et al., 2021). Leaves from seedlings grown on a medium were collected and ground to powder in liquid nitrogen. The powder was resuspended in 80% (v/v) acetone on ice and centrifuged at 10,000 g at 4°C for 5 min. A664 and A648 were inspected by spectroscopy absorbance measurements, and the chlorophyll a and b contents were calculated according to those described previously. NBT and DAB staining methods refer to (Zhang et al., 2011). For NBT staining, the leaves were soaked in darkness with 1 mg/mL nitro blue tetrazole (pH 7.8) for 40-60 min. For DAB staining, the leaves were washed with 1 mg/mL diaminobenzidine (pH 7.0) in the dark for 8 h. The leaves were then boiled in an ethanol glycerin (3:1) solution for 20 min and photographed. Evans Blue staining was performed as described by (Baker and Mock, 1994). Root damage was evaluated by staining with Evans blue solution (0.25%, w/v), The leaves were then boiled in an ethanol glycerin (3:1) solution for 20 min and photographed. H_2_O_2_ content was determined according to Elstner, 1976 and the specific procedures were carried out as per the manufacturer’s instructions. The tissues were homogenized in an ice bath with 0.1% trichloroacetic acid (TCA) and centrifuged at 12,000 ×g for 15 min. The reaction solution contained 0.5 mL of potassium dihydrogen phosphate buffer (pH 7.0), 1 mL of 1 M potassium iodide (KI), and 0.5 mL of supernatant. Following that, the mixture was kept at 25°C for 1 h and its absorbance was measured at the wavelength of 390 nm. The H_2_O_2_ content was calculated according to the standard curve obtained using different concentrations of H_2_O_2_. O_2_^−^ content was determined according to Elstner, 1976 and the specific procedures were carried out as per the manufacturer’s instructions. The O_2_^−^ content was determined by monitoring the absorbance of azo compounds at 530 nm (Willekens et al., 1997). The malondialdehyde (MDA) content of the detailed experimental method refers to (Puckette et al., 2007). Approximately 0.1 g plant tissue was homogenized in 1.5 mL 5% TCA that contained 0.25% TBA, incubated at 100°C for 30 min, cooled to room temperature, and then centrifuged at 15000 ×g for 10 min. The concentration of TBA-reactive substance was then determined by measuring the absorbance of the resulting supernatant at 440, 532, and 600 nm. SOD activity was determined refer to (Giannopolitis et al., 1977) by monitoring the absorbance of blue formazan generated by the reaction of the remaining O_2_^−^ with nitro-blue tetrazolium at 560 nm. The Catalase (CAT) activity was determined refer to (Soydam Aydin et al., 2013), tested in potassium phosphate buffer (pH 7.8) containing 3 mM H_2_O_2_ at 240 nm. The glutathione-s-transferase (GST) activity was determined using a previously described method (Nagalakshmi and Prasad, 2001). The reaction mixture contained 10 μL of 0.1 M 1-chloro-2,4-dinitrobenzene (CDNB), 100 μL of 10 mM GSH, 500 μL of 0.2 M KPO4 buffer, and 390 μL of distilled water. and the increase in absorbance was measured at the wavelength of 340 nm. One unit of GST was defined as the amount of enzyme that increased the absorbance by a unit of 1 per min, at the wavelength of 340 nm. Ferric-chelate reductase activity was determined using a previously described method (Li et al., 2023). The roots were immerged into Fe (III) reductase detection solution (0.1 mM Fe (III)-EDTA and 0.3 mM ferrozine), and 10 intact plants were used in each independent experiment. The reaction solution was placed in darkness at 22°C, and then the supernatant was analyzed spectrophotometrically at 562 nm. Lastly, the FCR activity was measured with the use of a molecular extinction coefficient of 28.6 mM^-1^cm^-1^.

### 2.8 Fe, Cd, Zn, Mn, and Cu concentration determination

The 13-day-old Arabidopsis seedlings were treated with or without 90 μM CdCl_2_ for 3 days. The roots and shoots were counted and harvested separately, and then washed with ddH_2_O 3~4 times. After being dried in a conventional oven at 70°C, the samples were digested completely in 65%~68% HNO_3_ at 120°C. The Fe, Cd, Zn, Mn, and Cu concentrations were determined as described by (Takahashi et al., 2019). Using inductively coupled plasma emission spectrometry ICP-OES8000 (Perkin Elmer, USA). The Fe, Cd, Zn, Mn, and Cu concentrations were calculated according to the method previously described (Wu et al., 2012).

### 2.9 Yeast one-hybrid assay

The *MsbHLH115* ORF sequence was cloned into the pGADT7 vector to construct the recombinant vector pGADT7-*MsbHLH115*. The E-box element sequence (CAAATG) and mutated E-box element sequence (ACAATG) were cloned into the pAbAi vector to form pE-box-AbAi and pmE-box-AbAi. The p53-AbAi+pGADT7-p53, pE-box-AbAi+pGADT7-*MsbHLH115*, and pmE-box-AbAi+pGADT7-*MsbHLH115* were transformed into yeast strain Y1H. the yeast was grown in SD/-Leu medium (Takara, Shanghai, China) containing 300 ng/mL AbA. The interaction was studied using a yeast single hybridization system (Clontech, Palo Alto, CA).

### 2.10 EMSA assay

The oligonucleotide probe E-box (CAAATG) synthesized the gene promoter and was labeled biotin at the 3’ end by Sangon (Shanghai, China). The *MsbHLH115* coding sequence was cloned into the pGEX4T-1 vector (provided by BioRun Biotechnology Co., Ltd, Wuhan, China), and EMSA analysis was performed using the photolytic chemiluminescence EMSA kit (Pierce, Rockford, IL, USA).

### 2.11 Dual-luciferase reporter assay

The *MsbHLH121* promoter was inserted into the pGreenII 0800-LUC (provided by BioRun Biotechnology Co., Ltd, Wuhan, China) to produce a report generator. The *MsbHLH115* ORF was inserted into pGreenII 62-SK to produce effectors. The mixture of the above plasmid fusion was injected into the lower epidermis of *Nicotiana benthamiana* leaves with a 1mL syringe without the needle. The infiltrated tobacco plants were cultured in low light for 2 days. Added 100 μL of freshly prepared Revilla substrate working liquid into the reaction solution, quickly mixed, and detected the Revilla luciferase activity immediately in the fluorescence detector.

### 2.12 Transient expression assay

pBI121-*MsbHLH121pro*::GUS and pBI121-3×E-Box::GUS recombinant vectors were constructed and transformed into Agrobacterium GV3101 by freeze-thaw method with pBI121-35S::GUS vector as the control. These vectors infiltrated into 3-week-old tobacco leaves, respectively. GUS activity was detected.

### 2.13 Statistical analysis

All of the data presented here were mean values for each treatment. There are at least three independent biological replicates in every experiment. SPSS 19.0 software and Origin 2018 software were utilized to perform the statistical analysis and produce the graphs, respectively. Student’s t-test was carried out between WT and transgenic plants in the same treatment, and one-way analysis of variance (ANOVA) followed by Duncan’s multiple range test was carried out between the control and treatments.

## 3 Results

### 3.1 *MsbHLH115* was responded to −Fe, Cd, and −Fe+Cd stress

**Figure 1:**
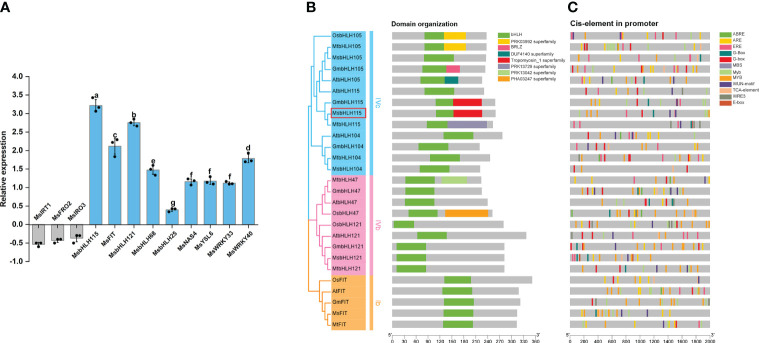
Bioinformatics, Expression level of Fe-deficient responsive genes under Cd stress. **(A)** Expression level of Fe-deficient responsive genes in the Alfalfa seedlings under Cd stress. Four-week-old alfalfa seedlings were treated with or without 90 μM CdCl_2_ for 3 days. *MsACTIN2* was the normalization control. The y-axis shows gene expression levels normalized to that of the CK. **(B)** The phylogenetic tree and structure domain of MsbHLH115 and other iron regulation bHLH genes. The MsbHLH115 protein of alfalfa was marked red. **(C)** Predicted cis-regulatory elements of bHLH promoter. Each type of cis-regulatory element is represented with different color box at the bottom. The five 5′ to 3′ direction represents the orientation of the nucleotide sequence, and the scale at the bottom represents the nucleotide length (bp). One-way ANOVA test. Mean ± standard error, n= 3. Lowercase letters indicate significant at *p*<0.05.

To explore the effect of Cd stress on Fe homeostasis, the expression levels of the genes involved in Fe-deficient response under Cd exposure in wild-type alfalfa. Compared with wild-type plants grown on standard medium, the results showed that *MsbHLH115*, *MsbHLH121*, *MsbHLH25*, *MsbHLH68*, *MsFIT*, *MsWRKY33*, *MsWRKY40*, *MsYSL6*, and *MsNAS* were significantly up-regulated; meanwhile, *MsFRO2*, *MsIRT1*, and *MsIRO3* were considerably down-regulated by Cd stress (*p*<0.05) (**Figure 1A**). Interestingly, *MsbHLH115* was most strongly induced by Cd at the transcription level, suggesting that *MsbHLH115* might be an essential gene involved in the network of Fe and Cd regulation in plants.

**Figure 2:**
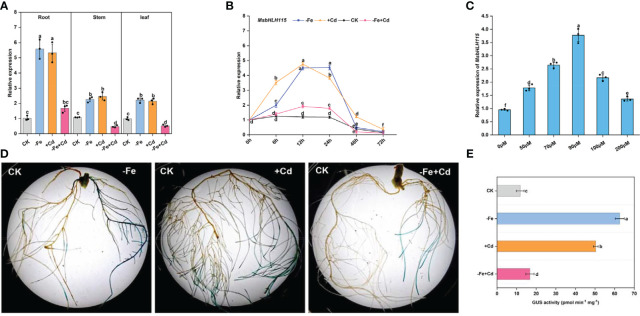
Expression pattern of *MsbHLH115*. **(A)** Expression level of *MsbHLH115* in different plant tissues. Four-week-old alfalfa seedlings were treated with −Fe, +Cd, and −Fe+Cd stress for 24 h. **(B)** Time-course expression level of *MsbHLH115*. Four-week-old alfalfa seedlings were treated with −Fe, +Cd, and −Fe+Cd stress for 6, 12, 24, 48, and 72 h. The expression level at 0 h was set as 1.0. **(C)** Expression level of *MsbHLH115* response to different concentration of Cd. Four-week-old alfalfa seedlings were treated with Cd. *MsACTIN2* was the normalization control. **(D)** Expression pattern of *MsbHLH115pro* under −Fe, +Cd, −Fe+Cd stress. **(E)** The *MsbHLH115pro* GUS activity measurement. One-way ANOVA test. Mean ± standard error, n= 3. Lowercase letters indicate significant at *p*<0.05.

*MsbHLH115* was expressed in roots, leaves, and stems of the alfalfa, mainly in roots. *MsbHLH115* was significantly up-regulated by −Fe and +Cd stress and considerably up-regulated by −Fe+Cd stress in roots, while down-regulated by −Fe+Cd stress in the stems and leaves of alfalfa (**Figure 2A**). The *MsbHLH115* expression was significantly increased by −Fe and +Cd at 6 h, 12 h, and 24 h, and was significantly down-regulated at 72 h. The *MsbHLH115* expression was significantly up-regulated by −Fe+Cd at 12 h and 24 h and was significantly down-regulated at 48 h and 72 h (**Figure 2B**). Following the increase of Cd concentration, *MsbHLH115* expression was significantly increased, reaching maximum expression at 90 µM, and then decreased (**Figure 2C**).

In the CK, the *MsbHLH115pro* transformed soybean hairy root showed β-D-glucuronidase (GUS) signals, indicating that the *MsbHLH115pro* had expression activity. The hairy root tip showed dark GUS staining in all treatments. Under −Fe and +Cd stress, the hairy root was darker blue. Under −Fe+Cd stress, the hairy root staining was darker than that in the CK but shallower than that in −Fe or +Cd treatment (**Figure 2D**). The value of GUS activity in soybean hairy roots is −Fe>+Cd>−Fe+Cd>CK (*p*<0.05) (**Figure 2E**). This result was consistent with the quantification of gene expression, indicating that *MsbHLH115* responds to −Fe, +Cd, and −Fe+Cd stress in plants.

### 3.2 Gene structure and promoter *cis*-element of *MsbHLH115* and its homologous genes

A total of 26 bHLHs containing typical bHLH domains were assigned to bHLHIVc, bHLHIVb, and bHLHIb subfamilies. Evolutionary analysis showed that MsbHLH115 belonged to the bHLHIVc subfamily. MsbHLH115 is most closely related to MtbHLH115, GmbHLH115, AtbHLH115, and AtbHLH105 (**Figure 1B**). There are some cis-regulatory elements such as G-box and E-box in the promoters of bHLH family genes. Several cis-elements involved in stress response and hormone regulation were found in the *MsbHLH115* promoter (**Figure 1C**).

### 3.3 MsbHLH115 localized in the nucleus

**Figure 3:**
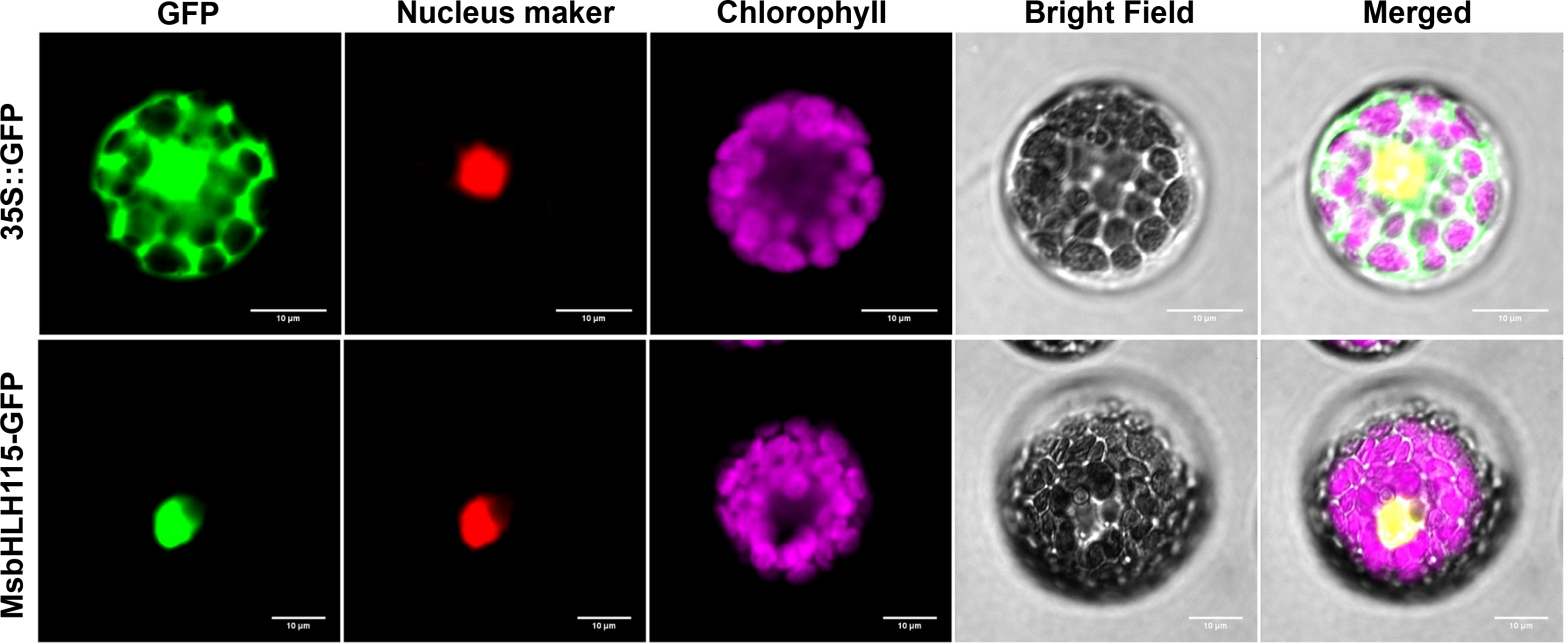
Subcellular localization of MsbHLH115. The 35S::GFP and 35S:: MsbHLH115-GFP plasmid were transformed in Arabidopsis protoplast cells respectively. Red fluorescence signals were used as a nuclear marker. Scale bars: 10 μm.

During the transient expression of the fusion protein in Arabidopsis protoplasts, green fluorescence was visible throughout the cytoplasm in protoplasts with the 35S:: GFP control. In protoplasts transiently expressing 35S::MsbHLH115-GFP, nuclear localization of the MsbHLH115-GFP fusion protein was revealed by a nuclear localization marker RFP. The alignment of the predominant red fluorescence in the nucleus with the GFP fluorescence signals confirmed the localization of MsbHLH115 in the nucleus (**Figure 3**).

### 3.4 Overexpression of *MsbHLH115* enhances Cd tolerance in Arabidopsis

**Figure 4:**
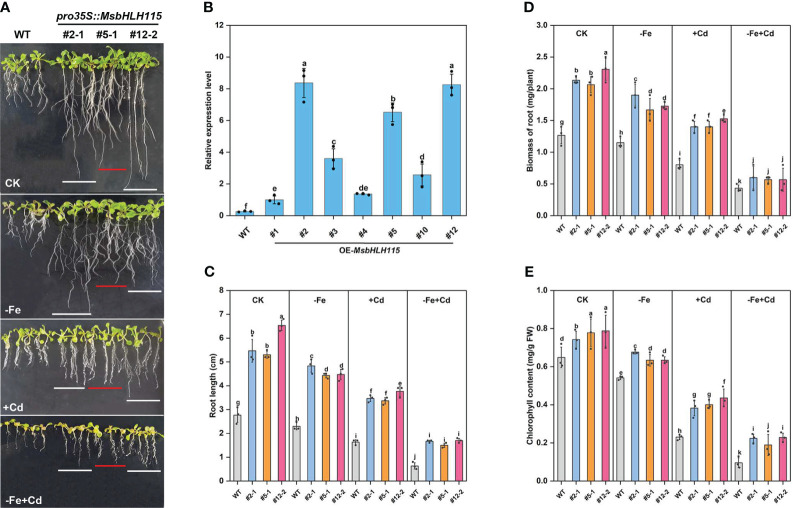
Determination of −Fe and Cd tolerance in overexpressed *MsbHLH115ox* T_3_ lines (ox#2-1, ox#5-1, and ox#12-2). **(A)** Phenotypes of WT and *MsbHLH115*ox Arabidopsis seedlings under −Fe, +Cd, −Fe+Cd stress and CK. **(B)** qRT-PCR in overexpressed *MsbHLH115ox* T_3_ lines. *MsACTIN2* was the normalization control. **(C)** Root length **(D)** Biomass of root. **(E)** The chlorophyll content. The 5-day-old seedlings were cultured on the 1/2 MS medium containing with/without Fe/Cd for 7 days. One-way ANOVA test. Mean ± standard error, n= 3. Lowercase letters indicate significant at *p*<0.05.

To investigate the biological function of *MsbHLH115*, we generated Arabidopsis overexpressed *MsbHLH115* lines (*MsbHLH115ox*). Three transgenic lines with high *MsbHLH115* expression levels (ox#2-1, ox#5-1, and ox#12-2) were selected for further analysis (**Figure 4B**). Under normal conditions(CK), −Fe, +Cd, and −Fe+Cd stress, the *MsbHLH115*ox lines showed significantly longer roots, higher biomass, and higher chlorophyll content than WT. The degree of impact on plant growth was −Fe+Cd >−Fe>+Cd(*p*<0.05) (**Figures 4A, C–E**). The severe growth inhibition caused by −Fe+Cd stress indicated that iron deficiency exacerbated the Cd toxicity in plants.

**Figure 5:**
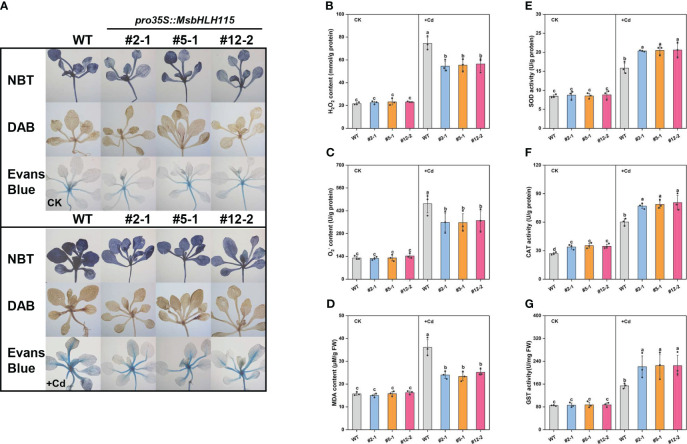
ROS and antioxidant enzyme activity in overexpressed *MsbHLH115ox* T_3_ lines (ox#2-1, ox#5-1, and ox#12-2) unde Cd stress. **(A)** NBT, DAB, and Evans blue staining. **(B)** H_2_O_2_ content. **(C)** O_2_^-^ content. **(D)** MDA content. **(E)** SOD activity. **(F)** CAT activity. **(G)** GST activity. The 5-day-old seedlings were cultured on the 1/2 MS medium containing Cd for 7 days. One-way ANOVA test. Mean ± standard error, n= 3. Lowercase letters indicate significant at *p*<0.05.

Under +Cd stress, the Nitro-Blue tetrazolium chloride (NBT), 3,3-diaminobenzidine (DAB), and Evans blue staining in the *MsbHLH115*ox leaves were lighter (**Figure 5A**) than WT; the O_2_^-^, H_2_O_2_, and MDA content in the three *MsbHLH115*ox lines was lower than WT (**Figures 5B–D**); the CAT, SOD, and GST activity of the three *MsbHLH115*ox lines were significantly higher than WT (**Figures 5E–G**). The result indicated that overexpression of *MsbHLH115* enhanced the tolerance of Arabidopsis to Cd stress and reduced the production of reactive oxygen species (ROS) in Arabidopsis under Cd stress.

### 3.5 Fe contributes to maintaining root growth under Cd stress

**Figure 6:**
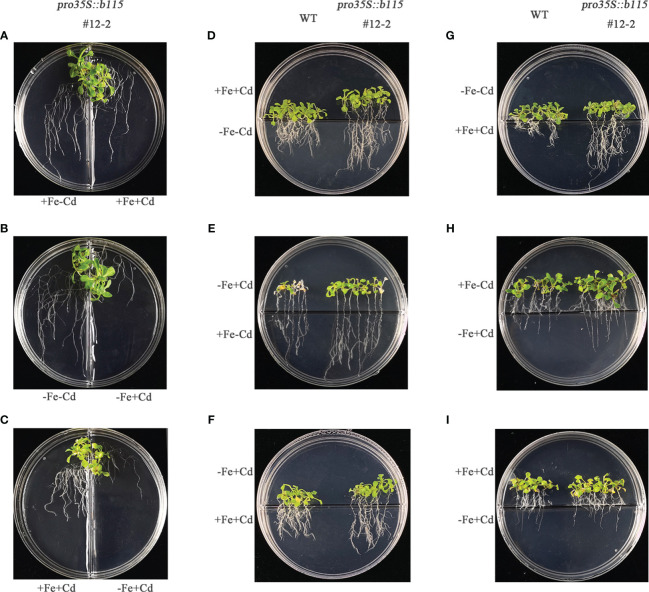
Effect of Fe status on the root elongation in *MsbHLH115ox* lines unde Cd stress. **(A–C)** The split-root experiment. The two roots were respectively transferred to different media (e.g., +Fe−Cd and +Fe+Cd, −Fe−Cd and −Fe+Cd or +Fe+Cd and −Fe+Cd) in one petri dish for 7 days when the cut-off-root seedlings were cultured on standard medium for 5 days. **(D–I)** The root-tip elongation experiment. The root tips and other parts of 5-day-old seedlings were exposed to the different media with/without Fe plus or minus Cd in one petri dish for 7 days.

Considering that Fe deprivation visibly elevated the susceptibility of transgenic plants to Cd stress, especially root growth, a split-root experiment was employed to further explore the impact of Fe status on Cd toxicity to plants. We first examined the effects of different Fe and Cd supplies on the growth of *MsbHLH115*ox line *#12-2*. In the split medium, the root growth of *#12-2* was inhibited by Cd under iron supply conditions (**Figure 6A**); the Cd inhibition of root growth was more obvious under iron deficiency (**Figure 6B**); and the Cd inhibition of plant root growth under iron deficiency was more obvious than that under Fe supply (**Figure 6C**). The result showed that the root elongation of *MsbHLH115*ox plants was inhibited by Cd, which was exacerbated by iron deficiency (**Figures 6A–C**, **Supplementary Figure S1**). We further compared the growth of *#12-2* and WT plants when the part near the root tip (RT) and the part distal the root tip (RS) was grown in different conditions. The *#12-2* root was longer than WT in the following cases, i. RT or RS was grown in Cd stress and the other part was stressed by iron deficiency (**Figures 6D, G**); ii. RT was grown in the −Fe+Cd stress and RS was grown in the +Fe−Cd medium (**Figures 6E, H**); and iii. RT was grown in Cd stress and RS was grown in the +Fe+Cd medium (**Figures 6F, I**). In other cases, the difference in growth of *#12-2* and WT plants was not obvious. The root elongation was affected by the total dose of Cd. In the presence of iron, *MsbHLH115* can rescue the root growth inhibition caused by Cd, whether the iron is in RT or RS; but in the absence of iron in the medium, especially in RT, the rescue function of *MsbHLH115* was negatively affected. The results showed that the iron in the rhizosphere played a leading role in maintaining root elongation under Cd stress.

### 3.6 Cd, Fe, Zn, Mn, and Cu accumulation in *MsbHLH115*ox plants

**Figure 7:**
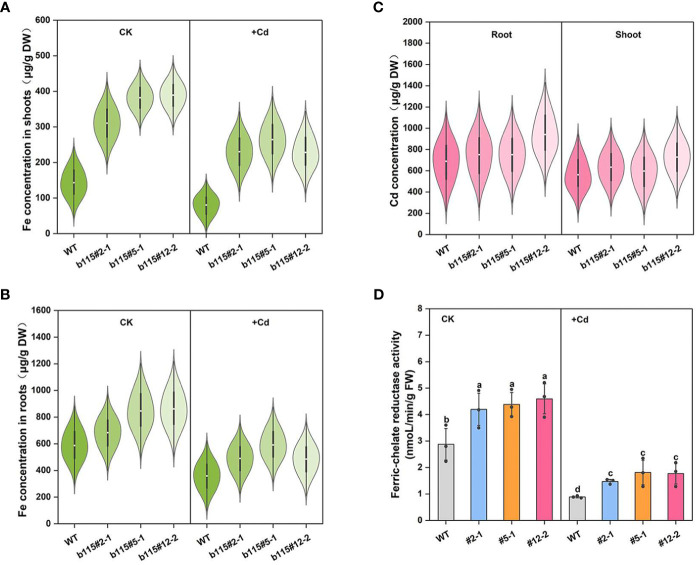
The Cd and Fe concentration and Ferric-chelate reductase activity in *MsbHLH115ox* lines under Cd stress. **(A)** Fe concentration in shoots. **(B)** Fe concentration in roots. **(C)** Cd concentration in the shoots and roots. **(D)** Ferric-chelate reductase activity. The 13-day-old seedlings were cultured on the 1/2 MS medium containing 0 or 90 μM CdCl_2_ for 3 days. Samples were taken for metal concentration determination and Ferric-chelate reductase activity determination. One-way ANOVA test. Mean ± standard error, n=3. Lowercase letters indicate significant at *p*<0.05.

The Fe concentration in the shoots and roots of three *MsbHLH115*ox lines was significantly higher than that in WT under both CK and Cd stress. The Fe concentration in shoots and roots of *MsbHLH115*ox lines and WT significantly decreased by Cd stress (**Figures 7A, B**). The Cd concentration showed no significant difference in he shoots and roots of *MsbHLH115*ox lines and WT. The results indicated that overexpression of *MsbHLH115* does not affect Cd absorption and translocation (**Figure 7C**). The Ferric-chelate reductase (FCR) activity in *MsbHLH115*ox lines was significantly higher than that in WT under CK and Cd stress (**Figure 7D**). The results indicated that *MsbHLH115* positively affects Fe accumulation and translocation in plants. Cd exposure reduced the plant’s ability to absorb Fe and decreased Fe accumulation in plants.

**Figure 8:**
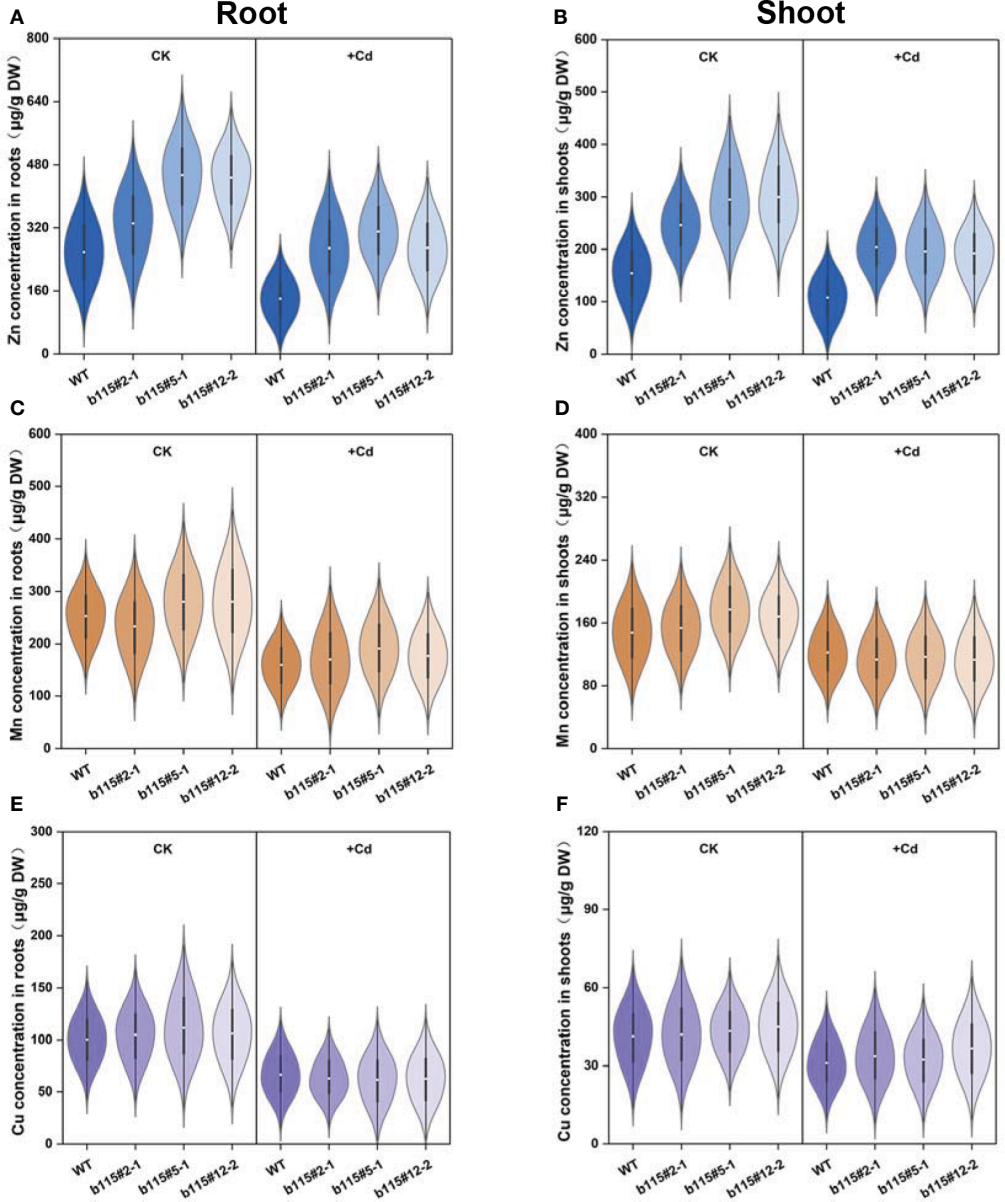
The Zn, Mn and Cu concentration in shoot and root in overexpressed *MsbHLH115ox* T_3_ lines (ox#2-1, ox#5-1, and ox#12-2) under Cd stress. **(A)** Zn concentration in roots. **(B)** Zn concentration in shoots. **(C)** Mn concentration in roots. **(D)** Mn concentration in shoots. **(E)** Cu concentration in roots. **(F)** Cu concentration in shoots. The 13-day-old seedlings were cultured on the 1/2 MS medium containing 0 or 90 μM CdCl_2_ for 3 days. Samples were taken for metal concentration determination. One-way ANOVA test. Mean ± standard error, n= 3.

Zn concentration in *MsbHLH115ox* was significantly higher than that in WT under Cd treatment or not. Cd stress significantly reduced Zn concentration in *MsbHLH115ox* lines and WT (**Figures 8A, B**). The results showed that Cd stress reduced Zn absorption, and led to a decrease in Zn accumulation in plants while *MsbHLH115* promotes Zn absorption and transport in plants. Mn and Cu concentration in *MsbHLH115ox* lines was higher than that in WT under Cd treatment or not, with no significant difference (**Figures 8C–F**). The results showed that Cd stress reduced Mn and Cu absorption and accumulation and *MsbHLH115* had little effect on Mn and Cu absorption and transport.

### 3.7 The expression of genes associated with Fe homeostasis, ROS-related and metal detoxification

**Figure 9:**
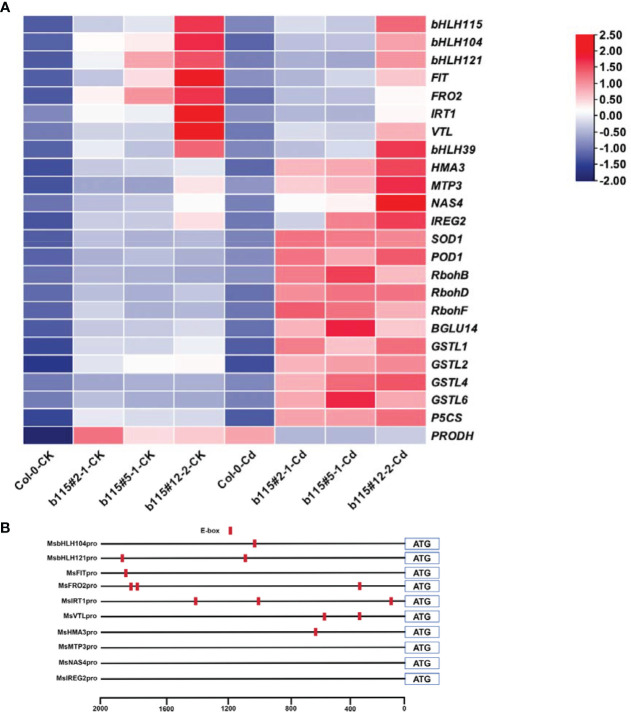
Expression level of various genes regulated by *MsbHLH115*. **(A)** Heatmaps showing the fold changes in expression of gene involved in Fe regulate genes (*bHLH115*, *bHLH39*, *FIT*, *bHLH121*, *bHLH104*). *IRT1*, involved in Fe uptake. *FRO2* is responsible for ferric reduction. *NAS4* is involved in Fe transport in the phloem. *HMA3* is involved in Cd transport in vacuoles. *IREG2* is involved in iron transport in chloroplasts. *MTP3*, involved in Fe transport. Glutathione genes (GSTL1, GSTL2, GSTL4, GSTL6). Proline synthesis and decomposition (P5CS, PRODH). Antioxidant genes (SOD1, POD1, and BGLU14). ROS-related genes (RbohB, RbohD, and RbohF). Boxes indicate relative expression level in genes. Log2 (fold changes) are represented by a color scale from white (down-regulated expression) to red (up-regulated expression). The 13-day-old seedlings were cultured on the 1/2 MS medium containing 0 or 90 μM CdCl_2_ for 24 h. *AtACTIN2* was the normalization control. **(B)** E-box elements of gene promoter. E-box was marked red.

As shown in **Figure 9**, the expression level of Fe uptake and translocation genes (*IRT1*, *FRO2*, *VTL*, *NAS4*, *IREG2*, *HMA3*, and *MTP3*) was higher in the three *MsbHLH115*ox lines than in the WT, regardless of whether they were under CK or Cd-exposed conditions. Under Cd stress, the expressions of *IRT1*, *FRO2*, and *VTL* expressions decreased, while the expressions of NAS4, HMA3, IREG2, and MTP3 increased in the *MsbHLH115*ox lines. The terms of these five transcription factors (*bHLH115*, *bHLH39*, *FIT*, *bHLH121*, and *bHLH104*) were higher in the *MsbHLH115*ox lines than in the WT and decreased by Cd stress. The expression of four *GSTs* was higher in the *MsbHLH115*ox lines and was enhanced by Cd stress. The expression of *RbohB*, *RbohD*, and *RbohF* was higher in the *MsbHLH115*ox lines and was enhanced by Cd stress. The expression of *SOD1*, *POD1*, and *BGLU14*, was higher in the *MsbHLH115*ox lines and was enhanced by Cd stress. The *P5CS* expression was higher in the *MsbHLH115*ox lines and was enhanced by Cd stress, while *PRODH* was higher in the *MsbHLH115*ox lines but was inhibited by Cd stress (**Figure 9A**). The promoters of *bHLH115*, *FIT*, *bHLH121*, *bHLH104*, *IRT1*, *FRO2*, *VTL*, and *HMA3* contain E-box elements, while *NAS4*, *IREG2*, and *MTP3* have no E-box element (**Figure 9B**).

### 3.8 The interaction of MsbHLH115 with E-box element and *MsbHLH121* promoter

**Figure 10:**
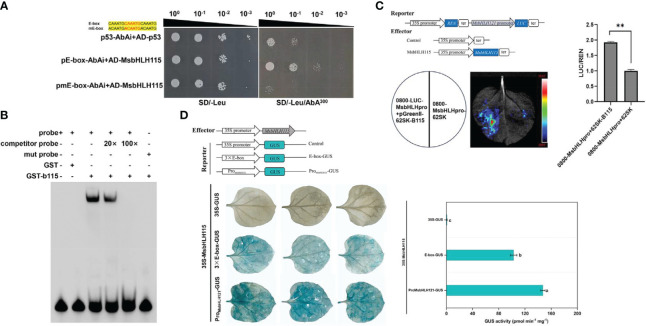
*MsbHLH115* interacted with E-box and *MsbHLH121*pro. **(A)** Yeast one-hybrid assays of *MsbHLH115* binding to the E-box. Constructs pGAD-Rec-p53 and p53-AbAi were used as positive control. Sequences of the *MsbHLH115* and corresponding mutant probes used in the electrophoresis mobility shift assay (EMSA). Yellow highlighting denotes the E-box and Mutated nucleotides. **(B)** EMSA assay of binding between *MsbHLH115* and E-box. The competitor probe was added at 20- and 100-fold more than the labeled probes respectively. A negative control was used to validate the EMSA system. **(C)** LUC assay of binding between *MsbHLH115* and *MsbHLH121pro*.The LUC/REN ratio represents the relative activity of the interaction activation. **(D)** Transient expression experiment showed that *MsbHLH115* interacted with E-box and *MsbHLH121pro*. GUS staining of representative leaf pieces infiltrated with coinfiltrated with the effector and the reporters. pBI121-*MsbHLH115* was used to the effector. pBI121-GUS, pBI121-*3×E-box*::GUS, pBI121-*MsbHLH121pro*::GUS was used to the reporters. GUS activity measurements were performed. One-way ANOVA test. Mean ± standard error, n= 3. Lowercase letters indicate significant at *p*<0.05. ** *p*<0.01.

On SD/-Leu medium without AbA, *p53*-AbAi+pGADT7-*MsbHLH115*, *pE-box*-AbAi +pGADT7-*MsbHLH115*, and *pmE-box*-AbAi+pGADT7-*MsbHLH115* transformed yeast strains can grow at diluted concentrations from 10^-1^ to 10^-3^. The background expression of *pE-box*-AbAi(linearized) in yeast was severely inhibited by 300 ng/mL AbA. On SD/-Leu medium supplemented with 300 ng/mL AbA, *p53*-AbAi+pGADT7-*MsbHLH115* and *pmE-box*-AbAi +pGADT7-*MsbHLH115* did not grow, while *pE-box*-AbAi+pGADT7-*MsbHLH115* could grow. The yeast one-hybrid (Y1H) assay showed that *MsbHLH115* can bind to E-box cis-element (**Figure 10A**). The results of EMSA analysis showed that there was a migration band between the E-box probe and the protein GST- *MsbHLH115*. The 20× competitive examination can compete with part of the migration band of the protein GST- *MsbHLH115*, and the 100× competitive examination can compete with most of the migration band of the probe and the protein GST- *MsbHLH115*. No migration band exists between the mutant probe and GST- *MsbHLH115*, and the E-box interacts with GST- *MsbHLH115*, further proving that the MsbHLH115 fusion protein can directly bind to the E-box (**Figure 10B**). LUC test showed that the enzyme activity of double luciferase was significantly higher than that of the control, indicating that MsbHLH115 could directly bind to *MsbHLH121* promoter (**Figure 10C**). The tobacco leaves that co-transformed pBI121-*MsbHLH115* and pBI121-*MsbHLH121pro*::GUS showed more blue spots than those that co-transformed pBI121-*MsbHLH115* and pBI121-*3×E-box*::GUS. However, no blue dots appeared in tobacco leaves of co-transformed pBI121-*MsbHLH115* and pBI121-GUS, indicating that MsbHLH115 protein can recognize E-box element and interact with *MsbHLH121* promoter (**Figure 10D**).

## 4 Discussion

The bHLH family members are essential in the regulation of plant iron homeostasis. Alfalfa *MsbHLH115* has a typical bHLH structural domain and is a homolog of *AtbHLH115*, which belongs to the bHLH IVc subfamily. The regulator of iron absorption, bHLH115, is a regulatory gene for *FIT* and *PYE*. However, little is known about the role of *bHLH115* in plant Cd tolerance. In this study, we carried out a series of experiments on *MsbHLH115* to elucidate whether *MsbHLH115* is a casual gene at the intersection of Fe and Cd stresses. *MsbHLH115* rapidly responded to Fe or Cd deficiency treatment at 6-24 hours, especially in the root of alfalfa (**Figure 2**). In previous transcriptome studies, 50 days or 42 days Cd treatment induced the expression of *FIT*, *PYE*, *bHLH38*, *bHLH39*, *IRT1*, *FRO2*, *NAS*, while *bHLH115* was not detected (Aprile et al., 2018). We found that *MsbHLH115* is an “early” response gene to Cd stress. Interestingly, the expression of *MsbHLH115* under −Fe+Cd combined stress was lower than that of −Fe or +Cd alone, indicating that the induced effects of −Fe and +Cd were non-cumulative and mutually inhibitory on *MsbHLH115*. This non-superposition or even mutual interference between −Fe and +Cd, together with the fact that the two metals Cd and Fe are antagonisms in plants, can be hypothesized that the function of *MsbHLH115* may differ in Cd and Fe stress.

Cd is toxic to plants, mainly impairing root growth and decreasing photosynthesis, which can be alleviated by overexpression of Cd resistance genes (Yao et al., 2018). Overexpression of *MsbHLH115* in Arabidopsis significantly improved these growth indicators, demonstrating that the *MsbHLH115* promoted plant growth and improved the plant tolerance to −Fe and Cd stress. Iron deficiency exacerbated the inhibition of plant growth by Cd, and under the −Fe+Cd complex pressure, although the Cd resistance indicator of the transgenic plants remained higher than WT, the growth advantage of the transgenic plants was significantly deprived (**Figure 2**). Split-site stress treatments revealed that *MsbHLH115* overexpression could reduce the toxic effects of Cd on plants, which is more effective in the presence of iron (**Figures 6A–C**). Similar to our results, previous studies have identified that the tolerance to Cd toxicity of an iron-regulating factor mutant *bts-1* was significantly reduced when iron was removed from the medium (Fan et al., 2020; Zhu et al., 2020). The results imply that *MsbHLH115* might enhance plant Cd tolerance by increasing iron uptake capacity. Regardless of Fe supply, Cd had a more significant effect on plant growth when exposure of seedling root tips and Fe supply in the root tips rescued the growth inhibition. The result coincides with the fact that the *MsbHLH115* promoter drives the expression of GUS in soybean hairy root tip under Cd stress, suggesting that the root tip is the primary tissue that *MsbHLH115* expresses in response to Fe and Cd. Thus, we inferred that *MsbHLH115* enhanced Cd tolerance, partly by increasing the iron uptake capacity.

*MsbHLH115* increased the Fe concentration, regardless of the presence of Cd, suggesting that *MsbHLH115* promotes the uptake and translocation of Fe in plants. Cd treatment induced up-regulation of *MxIRT1*, *MxFRO2-Like*, and *NtIRT1* (Hodoshima et al., 2007; Gao et al., 2011). These studies concluded that +Cd and −Fe regulate *IRT1* expression similarly and suggested that up-regulation of *IRT1* could prevent Cd-induced Fe deficiency (Sebastian and Prasad, 2018). Guan et al. concluded that inhibition of *IRT1* could reduce Cd uptake by roots (Guan et al., 2019). The up-regulation of *FRO2* and *IRT1* expression can maintain the high Fe concentration and enhance the tolerance in plants to Cd toxicity(Fan et al., 2020; Zhu et al., 2020). Ferric chelate reductase is required for iron metabolism in plant roots and shoots (Li et al., 2004; Wu et al., 2005). Recently, reduction in Fe reductase activity by Si application to Cd stress in Alfalfa (Kabir et al., 2016). In *SlbHLH068* VIGS plants, ferric-chelate reductase reduction in shoots, the expression of *LeFRO1*, and iron accumulation in leaves and roots were significantly diminished compared with control plants (Du et al., 2015). Two iron uptake genes, *IRT1* and *FRO2*, were significantly up-regulated by the *MsbHLH115*, which might be the reason for Fe accumulation in transgenic plants. These results suggest that FRO2 and IRT1 might maintain high iron concentrations, thereby reducing Cd toxicity through competition in *MsbHLH115* transgenic plants.

Metal translocation in plants is finely regulated by transporter proteins such as VTL, HMA3, MTP3, IREG2, and NAS4. Overexpression of *MsbHLH115* induced the expression of these genes. VTL plays an essential role in iron homeostasis in plants, storing excess iron in vesicles for slow release by plants (Ram et al., 2021). In *MsbHLH115*ox Arabidopsis, Cd stress down-regulates *VTL* expression might allow iron not to be bound to the vesicle but to be involved in the organism and used in functional organelles. *IREG2*, *NAS4*, *HMA3*, and *MTP3* are weakly responsive to Cd, whereas the inducible effects of *MsbHLH115* and Cd are cumulative for these genes. *NAS4* catalyze the formation of nicotinamide and increases Cd tolerance (Koen et al., 2013). *HMA3* and *MTP3* are involved in the segregation, retardation, chelation, and detoxification of heavy metals and are commonly used as indicator genes for plant resistance to Cd stress (Morel et al., 2009; Ueno et al., 2010; Qiang et al., 2017; Liu et al., 2018). These genes were positively regulated by Cd tolerant TFs *ANAC004*, *bHLH104*, *FIT*, and *AtbHLH38* or *AtbHLH39*, and *ZAT10* TFs (Wu et al., 2012; Dang et al., 2022; Meng Y. et al., 2022), as well as up-regulated by *MsbHLH115*, suggesting that *MsbHLH115*, like the above TFs, has a role in enhancing Cd tolerance by regulating the metal transporter genes in plants.

*MsbHLH115* up-regulates the expression of TFs, including the endogenous *AtbHLH115, bHLH104*, and *bHLH121* in Arabidopsis, but Cd stress impairs this up-regulatory effect. As a hub gene in the regulatory network, *FIT* interacts with *bHLH38*/*39* and *ZAT10* to up-regulate the expression of *IRT1* and *FRO2*, which helps plants take up iron and also plays a role in Cd tolerance (Wu et al., 2012; Dang et al., 2022). The weakening of the up-regulation of *FIT* and *AtbHLH39* genes in Cd-stressed trans-*MsbHLH115* plants may be responsible for the reduced expression of *IRT1* and *FRO2* and, subsequently, the reduced Fe concentration.

Cd accumulation leads to oxidative damage in plants. This study reduced ROS accumulation in *MsbHLH115*ox plants under Cd stress, similar to the performance of *AtbHLH104* and *AtILNF-YC6* overexpression plants. Increasing SOD, POD, and CAT enzyme activity helped to scavenge oxygen radicals in *MsbHLH115*ox plants. ROS-related genes (*RbohB, RbohD, RbohF*)*, SOD1*, and *BGLU14* were up-regulated at the transcriptional level, suggesting that *MsbHLH115* participates in Cd toxicity tolerance. In addition, GSH scavenges H_2_O_2_, thereby reducing Cd-induced oxidative stress in cells. Previous studies have found that *PyWRKY48* can promote Cd detoxification in plants by regulating GSH synthesis and GST expression (Wu et al., 2023). In this study, GST activity increased, and *GST*s up-regulated in *MsbHLH115*ox plants under Cd stress, suggesting that *MsbHLH115* is involved in Cd detoxification by inducing GST expression and up-regulating GSH synthesis (Sebastian and Prasad, 2018). Proline is one of the plants’ most critical abiotic osmolytes (Kim and Nam, 2013). *P5CS* gene was up-regulated, and *PRODH* was down-regulated in *MsbHLH115*ox plants under Cd stress, suggesting that *MsbHLH115* promotes proline synthesis and inhibits its catabolism, resulting in the accumulation of proline in the plant (Cecchini et al., 2011; Funck et al., 2012). Under Fe-deficient conditions, Cd-induced oxidative damage in *MsbHLH115*ox plants was still lower than in WT. Combined with the gene expression results, we hypothesized that *MsbHLH115* could also increase Cd tolerance in plants by regulating the expression of oxidase and detoxification genes in a Fe-independent manner. Heavy metals can affect photosynthesis and water metabolism in plants, resulting in wilting of leaves and slow growth (Xuan et al., 2016; Ma et al., 2017; Bashir et al., 2020). Cd may interfere with the plant’s water absorption and transpiration, causing the plant to be unable to absorb and utilize water effectively, thereby triggering drought stress (Xia et al., 2015). Several studies mentioned that plant biomass decreased due to water stress and Cd toxicity (Bashir et al., 2019; Khan et al., 2019). *MsbHLH115* might also alleviate drought stress caused by Cd by regulating antioxidant genes, thereby improving plant growth, although not confirmed by experiment in this study.

We found most *MsbHLHs* promoters contained E-box element, which could be bind to MsbHLH115. The instantaneous expression analysis and EMSA provide strong evidence to support the direct and specific interaction between the MsbHLH115 and *MsbHLH121* promoter. These data lead us to speculate that *MsbHLH121* is the target gene of *MsbHLH115*, suggesting that *MsbHLH121* may be assigned to bHLH to participate in Cd regulation. *bHLH121* controls the expression of many iron-deficiency response genes (Kim et al., 2019; Gao et al., 2020). Recent studies have shown that *IMAs* increase iron content and Cd tolerance in plants by activating iron absorption regulatory networks (Lei et al., 2020). *MsbHLH115* may also regulate other genes such as *FIT* and other bHLHs which we will examine in future work to construct the molecular regulatory network of cadmium tolerance. We will further elucidate the function of *MsbHLH115* by homologous transformation or inhibition of expression in alfalfa. Interestingly, the expression of the antioxidant genes was increased when *MsbHLH115* overexpressed in plants under Cd stress, which is also worth studying. There is also a strong possibility that some *MsbHLH115* controlled transporter genes, different from *IRT1*, transport Fe but not Cd. These candidate genes are looking forward to obtaining for the improvement of the Cd tolerance of plants.

## 5 Conclusion

In summary, we have identified a novel *MsbHLH115* transcription factor, which regulates Cd tolerance in Arabidopsis. Overexpression of *MsbHLH115* leads to iron accumulation increasing and downstream gene activation in Arabidopsis. Furthermore, *MsbHLH115* interacts with the E-box element. We also demonstrated direct and specific interactions between *MsbHLH115* and the promoter of *MsbHLH121*. Taken together, our findings suggest that *MsbHLH115* might regulate Cd tolerance by controlling the expression of the iron-regulate transcript factor *bHLHs* which increases iron transport in plants.

## Data availability statement

The original contributions presented in the study are included in the article/**Supplementary Material**. further inquiries can be directed to the corresponding authors.

## Author contributions

MZ: Conceptualization, Formal analysis, Investigation, Methodology, Validation, Writing – original draft, Writing – review & editing. J-YG: Conceptualization, Supervision, Writing – review & editing. S-CD: Conceptualization, Supervision, Writing – review & editing. M-HC: Conceptualization, Supervision, Writing – review & editing. J-XZ: Conceptualization, Supervision, Writing – review & editing. D-LG: Conceptualization, Resources, Writing – review & editing, Funding acquisition. C-HG: Funding acquisition, Resources, Writing – review & editing. Y-DB: Funding acquisition, Resources, Writing – review & editing.
