# Supplementary material for: Alfalfa MsbHLH115 confers tolerance to cadmium stress through activating the iron deficiency response in Arabidopsis thaliana
Source: Front Plant Sci. 2024 Feb 12;15:1358673. doi: 10.3389/fpls.2024.1358673 (PMC10894947; doi:10.3389/fpls.2024.1358673)
Supplement: Supplementary file 1 [file DataSheet_1.docx]

Supplementary Material

# Supplementary Data

Supplementary Material should be uploaded separately on submission. Please include any supplementary data, figures and/or tables.

Supplementary material is not typeset so please ensure that all information is clearly presented, the appropriate caption is included in the file and not in the manuscript, and that the style conforms to the rest of the article.

# Supplementary Figures and Tables

For more information on Supplementary Material and for details on the different file types accepted, please see [here](https://www.frontiersin.org/guidelines/author-guidelines#supplementary-material).

## Supplementary Figures


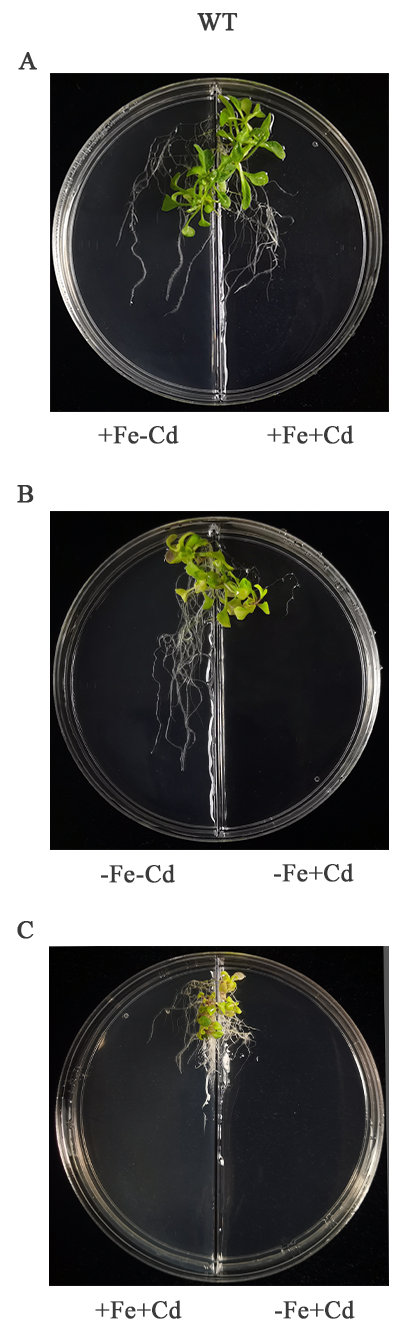


**Supplementary Figure 1.** The split-root experiment of wild type


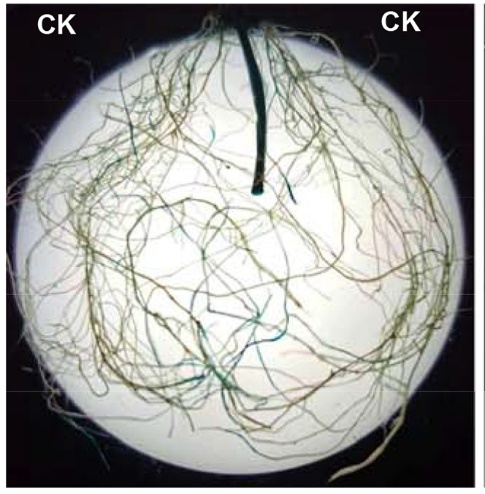


**Supplementary Figure 2.** Expression pattern of *MsbHLH115pro* under CK.


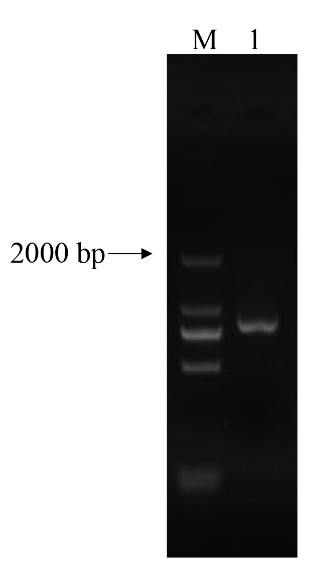


**Supplementary Figure 2.** Agarose gel electrophoresis detection. Acquisition of *MsbHLH115* gene from alfalfa. M： Maker DL 2000 1：PCR amplification of *MsbHLH115* gene.


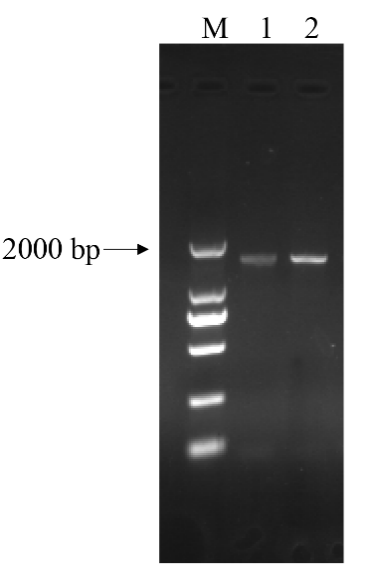


**Supplementary Figure 3.** Agarose gel electrophoresis detection. Acquisition of *MsbHLH115* gene promoter from alfalfa M: Maker DL 2000. 1-2：PCR amplification of *MsbHLH115* promoter.


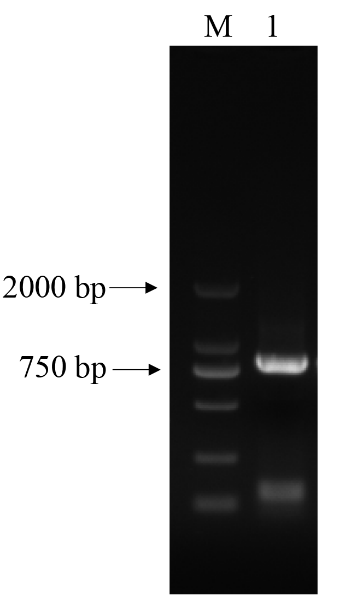


**Supplementary Figure 4.** Agarose gel electrophoresis detection. PCR amplification of the *MsbHLH115* gene removes the stop codon M： Maker DL 2000. 1： *MsbHLH115*.


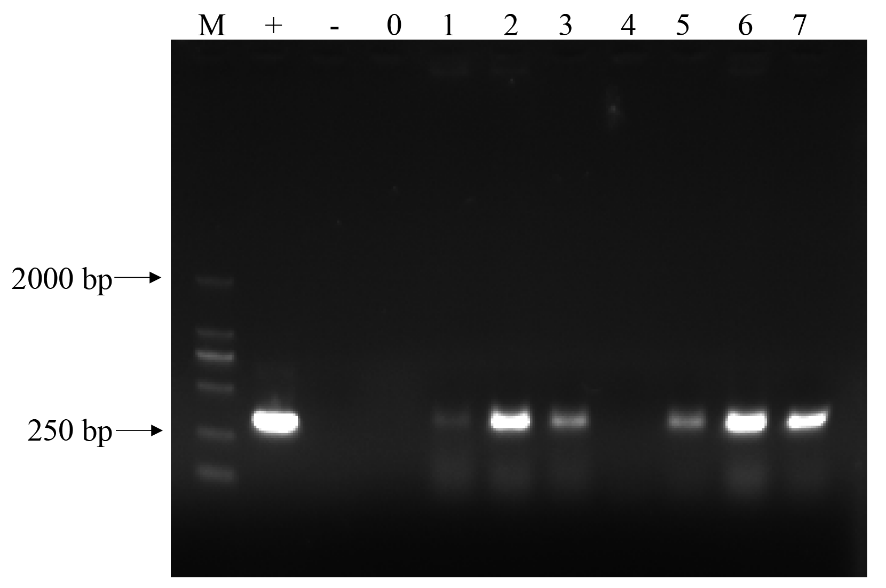


**Supplementary Figure 5.** The overexpressing *MsbHLH115* Arabidopsis detection by PCR analysis. a.1-7: transgenic Arabidopsis; +: positive control; -: Negative control；0: Blank control; M: Maker DL 2000.


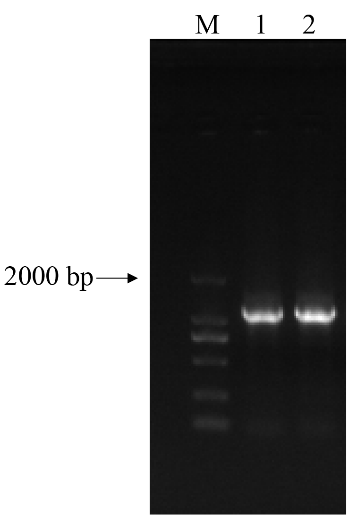


**Supplementary Figure 6.** Agarose gel electrophoresis detection. Acquisition of *MsbHLH121* gene promoter from alfalfa, M: Maker DL 2000. 1-2：PCR amplification of *MsbHLH121* promoter.


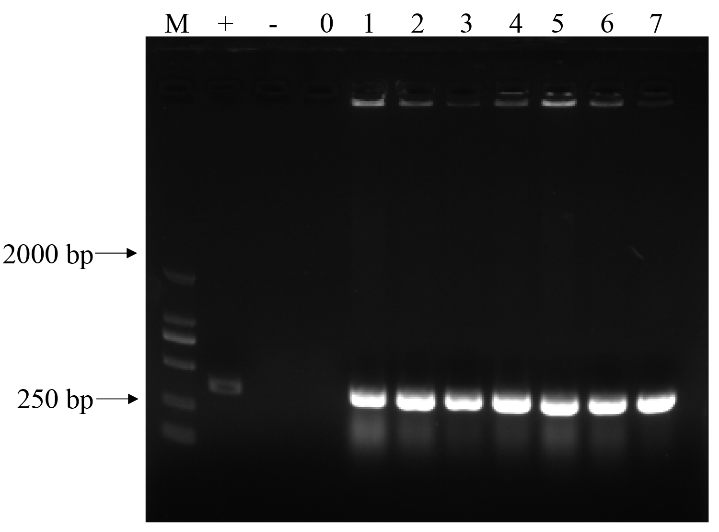


**Supplementary Figure 7.** PCR identification of pAbAi-E-box Vector. a.1-7: Positive colonies; + : E-box positive control; -: pAbAi Vector；0: Blank control; M: Maker DL 2000.


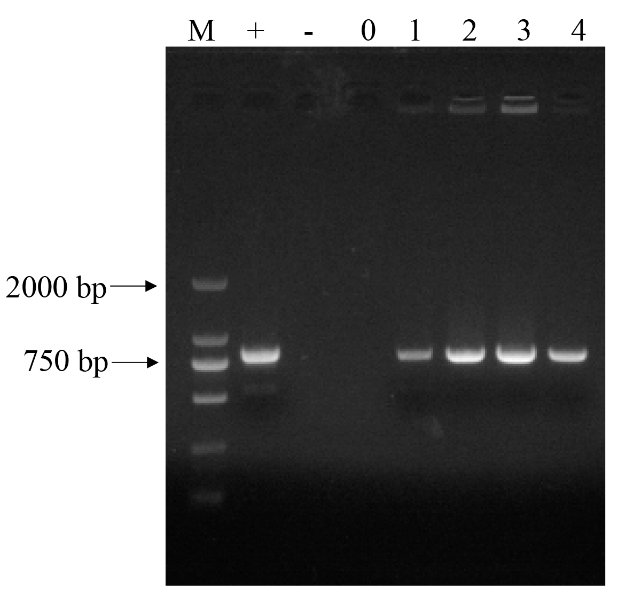


**Supplementary Figure 8.** PCR identification of AD-*MsbHLH115* Vector. a.1-4: Positive colonies; + : *MsbHLH115* positive control; -: pGADT7 Vector；0: Blank control; M: Maker DL 2000.


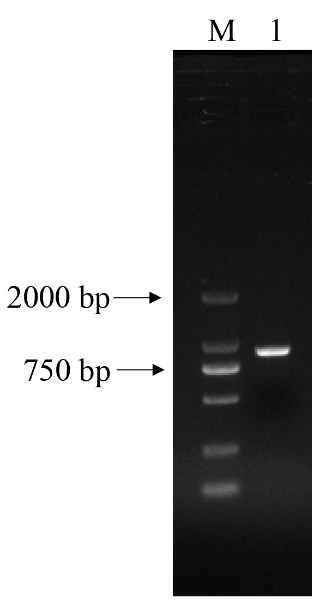


**Supplementary Figure 9.** Agarose gel electrophoresis detection. a. PCR amplification of *MsbHLH121* gene. M: Maker DL 2000. 1： *MsbHLH121.*

## Supplementary Tables

| Primer Comments | Primer sequence |
| --- | --- |
| MsbHLH115 cloning | F: CGGAATTCATGGATGATTCCAC |
|  | R:CGGGATCCTTAAGCAACTGGAGGGTC |
| qRT-PCR of MsbHLH115 | F: AACCCTCCTTCCTCCCATATG |
|  | R: TCGCACTTTCTCACGACCTG |
| MsbHLH115 GFPcloning | F: GCTCTAGAATGGATATGGATTCCACAG |
|  | R: CCCCCGGGAGCAACTGGAGGTCGAAG |
| MsbHLH115pro fragment cloning | F: CGGGATCCGGGTTGACAATAAGGGTG |
|  | R: GAAGATCTTGGGGTTCAACTTCAGAC |
| MsbHLH121 gene cloning | F:GCTCTAGAATGGAAATTACACCTGCTAG |
|  | R:CGGGATCCTCATTCACTAGCCTTTCTG |
| qRT-PCR of MsbHLH121 | F: AAGATTGTGGCTTCGGAT |
|  | R:TTCACTCCTACGCATACCA |
| qRT-PCR of MsFIT | F: ACTTGAAGCATCTTTATCAGTGTCT |
|  | R: AAAAGTGTCACAAACTGTAGCCAAG |
| qRT-PCR of MsbHLH68 | F: GGTGTTGTTCAAAGCTCTCCAG |
|  | R: TCATACCAATCTTATCTTCCTCCC |
| qRT-PCR of MsbHLH25 | F: AAAAGTGAAACTATTGGAGGAAAAG |
|  | R: AATGGACACGAATCAGCACG |
| qRT-PCR of MsWRKY33 | F: GAGTATGATTTCTTGTGTTGCCTTT |
|  | R: CATCTTCCACTCTCAAAGTTCCTAT |
| qRT-PCR of MsWRKY40 | F: AAGTGTAGAAGACCCGAGCAT |
|  | R: ACTAATAACTGCTGGATAGACGACT |
| qRT-PCR of MsYSL6 | F: CTCAGTCACAACGGGAAGG |
|  | R: TGGAACAGCCACAGCAAT |
| qRT-PCR of MsNAS4 | F: AAGATTGTGGCTTCGGAT |
|  | R: TTCACTCCTACGCATACCA |
| qRT-PCR of MsIRT1 | F:AGAGTGAGGTAACACCAGTG |
|  | R:TTAGGACTTTCTGAAGCACC |
| qRT-PCR of MsFRO2 | F:CACATCAAAATCAGGCAGAG |
|  | R:AGCAGTGAAAAGTGTCTAAGCA |
| qRT-PCR of MsIRO3 | F:ATTCTGCTCTTGGTGAAGTTGTA |
|  | R:CCTTTGTTTGCTCAGTGATTTT |
| qRT-PCR of AtbHLH115 | F: ACGAGCGTTTCAGATG |
|  | R: ACCTCCGATCCAGACA |
| qRT-PCR of AtbHLH121 | F: AACTTCTCCAAGAGTAAAGGTTTAAGA |
|  | R: AAGACGATCGACAGAATTAGGG |
| qRT-PCR of AtFIT | F: ACCCGCTGTTCCTGATAC |
|  | R: CCGAACCCCATACTGTTGTAAT |
| qRT-PCR of AtbHLH104 | F: GTAGCCTTTTTGTGATCGTC |
|  | R: CTTTCTGGAATTACACCAGC |
| qRT-PCR of AtIRT1 | F: AACCCTCCTTCCTCCCATATG |
|  | R: TCGCACTTTCTCACGACCTG |
| qRT-PCR of AtFRO2 | F: ACTTGAAGCATCTTTATCAGTGTCT |
|  | R: AAAAGTGTCACAAACTGTAGCCAAG |
| qRT-PCR of AtVTL | F: TGGATAATCTTACTGGCGTCCT |
|  | R: CCGAACCCCATACTGTTGTAAT |
| qRT-PCR of AtHMA3 | F: CCAGCTGTTGTTGTGTCAGC |
|  | R: GACCACAGGGACAACCACTT |
| qRT-PCR of AtMTP3 | F: CCACGAGAGATTGACCCGAC |
|  | R: TATCTGCCTCTGCCTCAGGT |
| qRT-PCR of AtNAS4 | F: ACACTTTTCAGCAATCTTAGGC |
|  | R: CTTTGGTCGGGACATGGGTT |
| qRT-PCR of AtIREG2 | F: TCACCTCTCAGAACCGGAGT |
|  | R: TAGAAGCAGCAACTCCAGCC |
| qRT-PCR of AtSOD1 | F: AAGAACAATAATTCTAAGAGAAGTG |
|  | R: CCTCGATCTTTGGGGTG |
| qRT-PCR of AtBGLU14 | F: AACCCTCCTTCCTCCCATATG |
|  | R: TCGCACTTTCTCACGACCTG |
| qRT-PCR of AtGSTL1 | F: GGAGATGAAGGAGGAGCTAT |
|  | R: ACTGCACGAGTCTTGAATTT |
| qRT-PCR of AtGSTL2 | F: GGCATCTGACCCTGACTTGTA |
|  | R: CGTCTGTTGGATGGTGGATAG |
| qRT-PCR of At*RbohB* | F: CCGTTGCAGGTTTGTCCGCAGTAG |
|  | R: TTGGCCCCAGCTGTCA |
| qRT-PCR of At*RbohD* | F: AATTTTATGTAGGCCACTGTGTAA |
|  | R: CCATCTTCGCAGCTACTG |
| qRT-PCR of At*RbohF* | F: TGGTTATGTAGGCTACTGCTGCTATG |
|  | R: CGAGATCCCACACTTGTG |
| qRT-PCR of AtGSTL4 | F: TGGTTATGTAGGCTACTGCTGCTATG |
|  | R: CCAGCCCAGGCCGTTG |
| qRT-PCR of AtGSTL6 | F: ACTGCCTTGTAATTTGTCACCCAT |
|  | R: TAATGGCATTCTTCTTCGCAGTAA |
| qRT-PCR of AtP5CS | F: AGCTCAATCTAAACCTGACTTGAAT |
|  | R: CTGGTACAATGACGCAAGGG |
| qRT-PCR of AtPRODH | F: TAAGTCCGACGCTGATGCTGAC |
|  | R: ATACTGGAACCGCTTAACCAGA |
| AD-MsbHLH115 fragment cloning | F: CGGAATTCATGGATGATTCCAC |
|  | R: CGGGATCCTTAAGCAACTGGAGGGTC |
| E-box | F: CCAAGCTTCAAATGCAAATGCAAATGCAAATG |
|  | R: CCCCCGGGCATTTGCATTTGCATTTGCATTTG |
| pAbAi | F: TGTGCTCCTTCCTTCGTTCT |
|  | R: ACATGGCAGTTTGGAGGTCT |
| pbI121-E-box-GUS | F: GGGGTACCCAAATGCAAATGCAAATGCAAATG |
|  | R: CCCCCGGGCATTTGCATTTGCATTTGCATTTG |
| EMSA1 | F:cagtGCCCGGGCtatggatatggattccacaggtggttcc |
|  | R: cgatGGTCTCattaagcaactggaggtcgaagcagg |
| EMSA2 | F: cagtGGTCTCattaatcgccatagcttgcatgcctgc |
|  | R: cagtGGTCTCaaccgtttttacggttcctggccttttgctgg |
| EMSA3 | F: gctggactgtacacttgct |
|  | R: cgcaattcccaatcttattcttgttct |
| pGreenII-MsbHLH115 | F: ATGGGTACAGAAACA |
|  | R: TCAGCTGCTTGCGGAA |
| pGreenII-ProMsbHLH121 | F: CGGGATCCGCTATCTATTATGTGCTATCT |
|  | R: CCAAGCTTGTTTGAGGTGAATCTGAG |
| MsbHLH121pro fragment cloning | F: GCTCTAGAGGGTTGACAATAAGGGTG |
|  | R: GAAGATCTTGGGGTTCAACTTCAGAC |
| MsACTIN | F: CCAAGGTCAAGATCGGAATCA |
|  | R: CAAAGCCACTCTAGCAACCAAA |
| AtACTIN2 | F: ACTTTCATCAGCCGTTTTGA |
|  | R: ACGATTGGTTGAATATCATCAG |
